# The effect of castration time on growth and carcass production of elk bulls

**DOI:** 10.1186/s40781-015-0072-2

**Published:** 2015-11-11

**Authors:** Sang-Woo Kim, Kwan-Woo Kim, Seong-Bok Park, Myung-Jick Kim, Dong-Gyun Yim

**Affiliations:** Animal Genetic Resources Center, National Institute of Animal Science, RDA, Namwon, 590-830 South Korea; Department of Public Health Administration and Food Hygiene, Jinju Health College, Jinju, 660-757 South Korea

**Keywords:** Elk, Castration time, Growth parameter, Carcass traits, Proximate composition

## Abstract

The effects of castration time on growth and carcass traits of elk bulls were investigated. Twelve bulls at 5 years old were raised and fed on concentrate with *ad libitum* hay. All animals were allocated randomly to each of four treatment groups (3 heads/group). Groups of each treatment were castrated surgically in March, April or June and managed together with non-castration (entire) treatment. All elk bulls in the trial were slaughtered at same time. Growth parameters, carcass yield and composition were recorded. The total gain and average daily gain was higher when castrated in April (*p* < 0.05). The entire elk produced heaviest and highest in saddle and brisket portions (*p* < 0.05). It is apparent that the castrate animals carried more total fat weight and percentages than the entire males (*p* < 0.05). It was found that loin muscles from non-castrated elk, in comparison with those from castrated one, had higher content of moisture and lower content of fat (*p* < 0.05). In this study, growth parameters, carcass yields and chemical composition were greatly affected by castration time.

## Background

The main products from deer production system are venison and velvet antler. Velvet antler is mainly consumed in Asian market including Korea as an ingredient of traditionally oriental medicine [1]. Venison, which means meat of deer, has high content of protein and minerals, as well as low fat, cholesterol and energy [2]. The essential feature in deer meat is that deer are very seasonally growing animals which gain weight rapidly in spring and summer and lose most of their fat during the autumn and winter period [3]. Although deer farming has elevated in Korea, it has several difficulties in the improvement of productivity due to little information about efficient feeding management [4].

The product of venison, velvet antler, skins and other minor components are all useful but in the long term the production of venison is likely to be the most important feature of deer farming [3]. Carcass composition is an important determinant of the animal's economic value, as different cuts have different commercial values [5]. The composition of primal cuts of carcass from deer grown in Korea has been little investigated.

The elk (*Cervus canadensis*) is one of the largest species of the Cervidae or deer family in the world and one of the largest land mammals in North America and eastern Asia [6]. In Korea, deer and elk has been imported from New Zealand and North America and raised about 50 years ago. The statistics show that 4,011 farm households have raised 48,463 head of deer and elk in Korea [7]. Although venison is not generally harvested for meat production on a large scale, some restaurants offer the meat as a healthy food in Korea. Venison is also a good source of minerals such as iron, phosphorus and zinc [8]. Several investigations have been made on red deer carcass such as slaughtering traits and carcass composition [5, 8], whilst the study on elk has been less studied. Castration could be a useful management practice if male deer are intended for venison production [5]. It may be particularly appropriate for bulls which will be slaughtered in the rut (breeding season) or shortly afterwards. Castration reduces the behavioural problems associated with managing entire stags during the rut, and carcasses from entire rusa stags slaughtered during the rut may have lower proportions of the more valuable joints than carcasses from castrates [5]. There is little information on the effect of castration time on the carcass traits in elk. Therefore, the aim of this study was conducted to assess the influence of castration time on growth and carcass traits from elk grown in Korea.

## Methods

### Animal feeding and castration procedure

Twelve elk bulls were born and raised at the Animal Genetic Resources Center, the National Institute of Animal Science (NIAS), Namwon, Korea. An experiment was undertaken with the approval of the NIAS, as required by NIAS regulation under the animal welfare. This experiment was carried out from February 2014 to October 2014. The fawns were weaned at 3 months. Concentrates and grass hay were offered ad libitum to each fawn until the onset of the experiment. When the real trial was started, the average age of elks estimated to be from 4 to 6 year of age. Chemical composition of experimental diets in elk bulls was shown in Table 1. Concentrates, consisting of corn, soybean meal, rapeseed meal, wheat bran, distillers dried grains and molasses, were provided twice a day with the amount of 1.8 % of body weight. Hay, which is a major roughage source for deer, was offered *ad libitum*. Animals were fed on mixed roughage (orchard grass, perennial rye grass, timothy, tall fescue, and kentucky bluegrass). Animals could drink freshwater any time in water buckets. Amount of feed and refusals were weighed daily to derive final weight, total gain and average daily gain.

**Table 1:** Chemical composition of experimental diets

Chemical composition	Concentrated feed	Hay
Moisture (%)	11.08	11.72
Crude Protein (%)	19.50	8.56
Crude Fat (%)	5.04	4.57
Crude Ash (%)	6.37	5.03
ADF(%)	9.68	30.76
NDF(%)	22.60	55.28

*ADF* Acid detergent fiber; *NDF* Neutral detergent fiber

Bulls at 5 year of age were surgically castrated either in mid-March, -April or -June. T4 animals were only left entire without castration to compare the effect of castration. Castration was occurred by surgical removal under general sedation and local anaesthesia. Animals were individually sedated by intravenous injection of 3 mL/head xylazine. After 2–3 min from injection, the scrotum was incised, the testes exteriorized and, following clamping of the cords and vessels, the testes were pulled out free. A subcutaneous injection of long-acting antibiotic solution (1 mL/10 kg live weight, Norocillin LA; Norbrook NZ Ltd, Auckland, NZ) and an intramuscular injection of 0.5 mg/kg meloxicam, a longacting analgesic and anti-inflammatory, (2.5 mL/100 kg live weight of Metacam 20; Boehringer Ingelheim NZ Ltd, Auckland) were delivered prior to reversal of general sedation by intravenous injection of 0.25 mg/kg yohimbine hydrochloride and 0.003 mg/kg naloxone hydrochloride (2.5 mL/100 kg live-weight of Contran H; Parnell Laboratories NZ Ltd, Auckland). Animals were held in a recovery pen for 1–3 h prior to being returned to pasture with herd-mates.

### Slaughter procedure

All animals were allocated randomly to each of four groups (3 heads/group) according to castration time: T1 (mid-Mar), T2 (mid-Apr), T3 (mid-Jun). T4 animals were entire (non-castration treatment). All bulls (5 year of age) were slaughtered at the NIAS slaughterhouse using standard procedures of NIAS in October 2014 when they were in the rut (breeding season). Following slaughter, the carcasses were held in chilled storage (4 °C) for a minimum 24 h prior to carcass dissection. Live weight gain was calculated as a difference between initial and final live weight over specified intervals. For carcass measurement, slaughter weight, cold carcass weight, dressing and fat percentage were measured using mechanical weighing balance (150 kg, HB Series, Korea). Carcass weights were obtained immediately following slaughter/dressing (hot carcass weight) and immediately before carcass dissection (cold carcass weight). Carcasses were broken down into primal joints and the weight of the primal cut was obtained.

### Proximate composition

For chemical composition, the *longissimus* muscle (LM) was excised from the saddle (strip loin or rib eye). All extraneous fat was manually removed from the loin muscles by a knife. All determinations were carried out on the homogenized sample, in triplicate. The proximate composition of meats was determined by a slightly modified method of the Association of Official Agricultural Chemists [9]. Moisture content was determined by drying 3 g of samples placed in aluminum dishes for 15 h at 104 °C. Crude protein content was measured by the Kjeldahl method (VAPO45, Gerhardt Ltd., Idar-Oberstein, Germany). The amount of nitrogen obtained was multiplied by 6.25 to calculate the crude protein content. Crude fat content was measured by the Soxhlet extraction system (TT 12/A, Gerhardt Ltd., Germany). Crude ash content was measured by burning 2 g of sample overnight in a furnace at 600 °C.

### Statistical methods

The experiment was conducted as 3 independent trials with 3 observations for each treatment. An analysis of variance (ANOVA) were performed on all the variables measured using the General Linear Model (GLM) procedure of the SAS statistical package [10]. Duncan’s multiple range test (*p* < 0.05) was used to determine differences between means. Mean values and standard deviations were reported.

## Result and discussion

Growth parameters of bulls according to the castration time are presented in Table 2. Significant differences were observed in total gain and average daily gain among the different castration time groups. Total gain and average daily gain of elk bulls were highest in T2 and lowest in T3 (*p* < 0.05). Commonly, deer have a strong seasonality in body weight and feed intake and these seasonal changes are probably consequences of an underlying seasonal rhythm in metabolic rate and a decreasing digestibility in winter [11]. Souma et al. [12] evaluated seasonal changes in feed intake of deer and observed that both the hay intake per day and daily gains of body weight increased from spring, reaching the maximum in summer, but decreased in autumn, reaching the minimum in winter. This is a common phenomenon largely attributed to low basal metabolic rate and feed intake [13].

**Table 2:** Growth parameters for the elk bulls according to the castration time

Castration time^a^	Initial weight (kg)	Final weight (kg)	Total gain (kg)	Average daily gain (kg/day)
T1	307.00 ± 35.59	347.33 ± 39.55	40.00 ± 4.41^bc^	0.21 ± 0.02^bc^
T2	320.33 ± 23.86	362.67 ± 21.94	42.67 ± 3.04^b^	0.22 ± 0.02^b^
T3	311.33 ± 40.80	334.67 ± 44.02	23.00 ± 5.05^d^	0.12 ± 0.03^d^
T4	305.00 ± 13.00	340.00 ± 14.00	35.10 ± 0.70^c^	0.18 ± 0.01^c^

All values are Mean ± SD (*n* = 3)

^a^T1: mid-Mar, T2: mid-Apr, T3: mid-Jun, T4: entires

^b-d^Figures with different letters within a same column differ significantly (*p* < 0.05)

Carcass yield and composition for the elk bulls according to the castration time is illustrated in Table 3. The mean live weight at slaughter ranged from 316 to 360 kg. The live weight and carcass weight of elk bulls was highest in T2, even though they were not significantly different. Hogg et al. [14] reported that deer growth and live weight could be variable as a result of different environmental influences. Asher et al. [15] exhibited a seasonal pattern of live weight changes, with greatest daily live weight change occurring in spring (especially in April) and live weight losses between May and July in New Zealand which is located at Southern hemisphere. In red deer, rutting bulls decrease in feed intake and lose live weight during the autumn season [5]. The overall mean dressing percentage ranged from 56.8 to 61.9 %, with no significant effect on castration time. Drew [3] noted that deer have dressing percentage in the range of 55–57 % and this is similar to the present study.

**Table 3:** Carcass yield and composition in elk bulls according to the castration time

	T1^a^	T2	T3	T4
Live weight at slaughter (kg)	324.40 ± 20.93	360.00 ± 19.52	322.80 ± 75.80	316.80 ± 14.14
Hot carcass weight (kg)	187.80 ± 20.01	211.70 ± 30.03	196.90 ± 20.01	201.20 ± 27.05
Cold carcass weight (kg)	184.20 ± 10.18	206.00 ± 23.76	197.10 ± 40.02	195.90 ± 0.99
Dressing weight (kg)	129.90 ± 10.02	153.23 ± 29.03	143.00 ± 30.03	156.41 ± 10.06
Dressing percentage (%)	56.80 ± 0.57	57.15 ± 3.46	61.30 ± 1.98	61.90 ± 2.40
Total meat weight (kg)	129.91 ± 10.60	153.24 ± 16.28	143.00 ± 33.52	157.42 ± 0.11
Total meat Percentage (%)	70.50 ± 1.84^b^	74.40 ± 0.71^c^	72.35 ± 2.33^c^	79.85 ± 0.35^b^
Total fat weight (kg)	25.25 ± 1.53^b^	23.72 ± 6.70^b^	24.55 ± 1.08^b^	10.53 ± 0.24^c^
Total fat Percentage (%)	13.75 ± 1.60^b^	11.40 ± 1.94^b^	12.66 ± 2.02^b^	5.38 ± 0.15^c^
Head weight (kg)	14.62 ± 6.07	17.19 ± 4.05	14.52 ± 5.01	16.04 ± 5.02
Velvet antler yields (kg)	8.80 ± 1.71	8.42 ± 1.23	9.83 ± 2.21	8.90 ± 0.14
Total Intestine weight (kg)	48.97 ± 5.04	51.69 ± 7.01	50.04 ± 8.02	37.51 ± 20.07
Bone weight (kg)	29.16 ± 1.33	28.90 ± 1.82	29.25 ± 1.23	29.00 ± 0.87
Hindquarter weight (kg)	6.49 ± 1.03	7.91 ± 1.64	7.12 ± 1.46	7.16 ± 1.77
Hindquarter percentage (%)	3.52 ± 0.53	3.84 ± 0.62	3.61 ± 0.22	3.65 ± 0.79
Forequarter weight (kg)	15.99 ± 5.04	18.12 ± 5.31	17.57 ± 4.03	19.39 ± 5.03
Forequarter percentage (%)	9.90 ± 0.93	8.68 ± 0.61	8.80 ± 0.53	8.91 ± 0.34
Saddle weight (kg)	19.53 ± 1.04^d^	23.67 ± 1.01^c^	20.87 ± 1.05^d^	26.34 ± 1.09^b^
Saddle percentage (%)	10.60 ± 0.41^c^	11.49 ± 0.65^c^	10.59 ± 0.98^c^	13.29 ± 0.14^b^
Tenderloin weight (kg)	3.73 ± 1.03	4.10 ± 1.01	3.98 ± 1.04	4.09 ± 1.44
Tenderloin percentage (%)	2.02 ± 0.74	1.99 ± 0.45	2.02 ± 0.61	2.09 ± 0.81
Brisket weight (kg)	14.37 ± 3.14^d^	16.94 ± 2.29^c^	16.63 ± 1.94^c^	20.26 ± 1.39^b^
Brisket percentage (%)	7.80 ± 1.23^c^	8.22 ± 0.89^c^	8.44 ± 1.09^c^	10.34 ± 0.39^b^
Rib weight (kg)	19.97 ± 4.33	23.14 ± 5.06	21.63 ± 4.54	19.06 ± 6.21
Rib percentage (%)	10.84 ± 2.03	11.23 ± 3.32	10.97 ± 3.01	9.73 ± 5.32
Chunk weight (kg)	7.47 ± 4.54	9.26 ± 5.87	8.42 ± 6.87	14.92 ± 4.64
Chunk percentage (%)	4.06 ± 4.43	4.49 ± 4.65	4.27 ± 5.84	7.62 ± 5.83

All values are Mean ± SD (*n* = 3)

^a^T1: mid-Mar, T2: mid-Apr, T3: mid-Jun, T4: entires

^b-d^Figures with different letters within a same row differ significantly (*p* < 0.05)

There was a significant castration effect on total meat percentage, fat weight and percentage. Total meat percentage of the entires was higher than those of the castrates, whereas total fat weight and percentage of the entires were lower than those of the castrates (*p* < 0.05). Similarly, previous studies [16, 17] demonstrated that castrated stags were lighter than intact stags. Castration in male animals has been widely shown to reduce growth rate and increase fatness when comparisons are made with entire males [18]. Surgical and chemical castration of deer has been demonstrated to affect animal live weight gain and carcass composition, mainly fat content [16, 19]. For saddle and brisket primal cuts, weight and percentage of the entires as the rut commences were higher than those of the castrates (*p* < 0.05) (Table 3). Drew [3] showed the castration of venison carcass increases the proportion of the saddle as rut approaches.

An analysis of the proximate composition of meat (Table 4) showed that the loin muscles of the entires had higher content of moisture and lower content of fat than those of the castrates (*p* < 0.05). The crude fat contents of bulls were highest when castrated in early time (T1) and lowest in entires (T4). These could be explained by the castration effect. Similar result noted that castration increases the fat content of the muscle yet decreases water content [17]. In a study by Dzierzynska-Cybulko and Fruzinski [20], differences in the proximate composition of deer meat could be related to various factors such as diet and seasonal variation. Moisture, crude protein and fat contents were in the range of 73–75 %, 22 % and 1–3 %, respectively. This is in agreement with Kim et al. [21] who reported that the moisture, protein and fat content of elk (*M. longissimus dorsi*) were 73.1, 22.4 and 2.11 %, respectively. According to Dzierzynska-Cybulko and Fruzinski [20], the protein and fat content of roe deer meat may range from 19.2 to 24 % and from 0.3 to 3.7 %, respectively. As demonstrated by Zomborszky et al. [22], the protein and fat content of roe deer meat (*M. longissimus dorsi*) was 23 and 1.7 %, respectively. Paulsen et al. [23] reported that the average levels of protein and fat in roe deer meat (fillet, leg, shoulder) ranged from 20.4 to 22.2 % and from 0.4 to 0.6 %, respectively. According to Hoffman and Wiklund [24], venison could fulfill the expectations and dietary requirements of the modern consumer, due to a low content of fat and high levels of protein and minerals. In our study, the high protein content and the low fat content in the elk meat obtained indicating a similar result compared to the findings of venison.

**Table 4:** Proximate composition of elk bulls according to the castration time

Castration time^a^	Moisture	Crude protein	Crude fat	Crude ash
	(%)			
T1	73.1 ± 0.56^c^	22.2 ± 0.13	3.00 ± 0.46^b^	0.83 ± 0.00
T2	73.8 ± 1.00^c^	22.2 ± 0.80	2.20 ± 0.28^c^	0.89 ± 0.04
T3	73.6 ± 0.58^c^	22.8 ± 0.84	2.01 ± 0.54^c^	0.93 ± 0.04
T4	75.7 ± 0.13^b^	22.0 ± 0.30	1.09 ± 0.33^d^	0.92 ± 0.06

All values are Mean ± SD (*n* = 3)

^a^T1: mid-Mar, T2: mid-Apr, T3: mid-Jun, T4: entires

^b-d^Figures with different letters within a same column differ significantly (*p* < 0.05)

## Conclusion

It was concluded that castration or castration time could affect the growth parameters, carcass yields and chemical composition. Results from this study demonstrate that total gain and average daily gain of elk bulls were highest when castrated in April and lowest in June. The stags in non-castration group had a notable exception. The results have shown that castration of bulls increased total meat percentage including saddle and brisket cuts compared to the entires and decreased total fat weight and percentage. Also, the castrates had lower content of moisture and higher content of fat than the entires elk. Further examination is required to identify castration effects on meat quality traits in bull. The result of this study could provide the guidelines that can be used to improve the carcass quality in old elk bull production along with velvet antler in Korea.
